# Effects of Non-Invasive Ventilation with different modalities in patients undergoing heart surgery: Protocol for a randomized controlled clinical trial

**DOI:** 10.1371/journal.pone.0304569

**Published:** 2024-06-18

**Authors:** Eder Rodrigues Araújo, Ivan Daniel Bezerra Nogueira, Paulo Eduardo e Silva Barbosa, Patrícia Angélica de Miranda Silva Nogueira

**Affiliations:** 1 Postgraduate Program in Physiotherapy (PPGFIS), Laboratory of Measures and Evaluation in Health, Federal University of Rio Grande do Norte (UFRN), Natal, Rio Grande do Norte, Brazil; 2 Department of Physical Therapy, Federal University of Rio Grande do Norte (UFRN), Natal, Rio Grande do Norte, Brazil; 3 Center of Technology Strategies in Health, State University of Paraiba, Campina Grande, Brazil; Kaohsuing Medical University Hospital, TAIWAN

## Abstract

**Introduction:**

The thoracic surgical procedure leads to a reduction in respiratory muscle strength. To restore it, certain strategies must be employed. Physiotherapy utilizes resources and techniques such as deep breathing stimulation, cough stimulation, use of incentive spirometers, mobilization, and ambulation. However, at times these resources and techniques may prove insufficient, and additional measures, such as Non-Invasive Ventilation (NIV), are employed Pieczkoski (2017). Non-Invasive Positive Pressure Ventilation (NPPV) has been utilized to expedite pulmonary function recovery as well as to prevent and treat postoperative pulmonary complications Nasrala 2018. NIV diminishes the risk of ventilator-associated complications due to its non-invasive nature. Consequently, NIV has been adopted to avert post-extubation complications in postoperative patients Liu 2020. The objective of this study is to conduct a randomized clinical trial and assess the efficacy of NIV in comparison to conventional physiotherapy in terms of pulmonary function among patients undergoing cardiac surgery at a selected hospital in Campina Grande, Paraíba, Brazil.

**Methods and analyses:**

This randomized, controlled, double-blind (patient and analyst) clinical trial will be conducted at Hospital João XXIII in Campina Grande, Paraíba, Brazil. Patients do not know which group they are allocated to. Those in the group that use CPAP or BIPAP will not be able to distinguish one from the other. The data analyst at the end of the collections will also be blinded. Only the health professional who will be applying the protocol cannot be blinded. The sample size, determined via sample calculation, yielded a total of 21 patients per group (63 patients). The patients will be allocated into 3 groups (CPAP group - CPAP + standard physiotherapy, BiPAP group - BiPAP + standard physiotherapy, and Control group - standard physiotherapy) in a 1:1:1 allocation ratio. The control group will receive the usual physiotherapeutic treatment as per the kinesiotherapy protocol. The treatment will be administered twice daily, starting in the ICU and progressing to the ward. In the CPAP group, nasal CPAP at 10cmH2O will be administered for 1 hour, twice daily, using an approved device. In the BiPAP group, nasal BiPAP with an IPAP of 13cmH2O and EPAP of 8cmH2O will be administered for 1 hour, twice daily, using an approved device. The NIV sessions will be conducted over the course of 5 days of hospitalization, both in the ICU and the ward. Assessments will be conducted at two time points: on day 1 preoperatively and on day 5 postoperatively. The following measures will be evaluated: pulmonary function, length of hospital stay, presence of postoperative pulmonary complications, score of the Minnesota Living with Heart Failure Questionnaire (MLHFQ) in its Portuguese version, functional capacity, the Global Perception of Change Scale, and the Functional Independence Measure (MIF). The normality of variables will be assessed using the Shapiro-Wilk test. IBM SPSS Statistics Base 25.0, using the Shapiro-Wilk test for normality and paired Student’s t-test for pre-post intervention comparison. They will use linear mixed effects models for longitudinal analysis and GLMMs to compare NIV effects over time between groups. They will employ ITT for missing data, INAR models for time dependence, fixed effects models for endogeneity, and Cohen’s d for effect sizes. Parametric model assumptions will be checked, and various models will be considered for data characteristics.

**Primary outcomes:**

Pulmonary function, Length of hospital stay.

**Second outcomes:**

Score of the Minnesota Living with Heart Failure Questionnaire (MLHFQ) in Portuguese version, Funcional capacity, The global perception of change scale, The functional independence measure (MIF), pO2 (partial pressure of oxygen), pCO2 (partial pressure of carbon dioxide), HCO3 (bicarbonate), Arterial Oxygen Saturation (SaO2), Base Excess (BE), Presence of lung complications.

**Other pre-specified outcomes:**

Duration of cardiopulmonary bypass, type of surgery, personal history, preoperative ejection fraction, previous respiratory complications, body mass index (BMI), gender and age.

**Trial registration:**

Trial register number NCT05966337.

## Introduction

Cardiovascular diseases are leading causes of both mortality and hospital admissions. Compared to other treatment modalities, cardiac surgery is distinguished by its advanced techniques and materials, which have led to safer procedures and reduced perioperative risks [1, 2].

In patients with cardiovascular diseases, including valvular disease, current guidelines advise considering exercise capacity for diagnosis and treatment planning [3, 4]. Moreover, coronary artery disease is the leading global cause of death, and patients undergoing coronary artery bypass surgery constitute a high-risk group [5]. Postoperative pulmonary dysfunction in patients undergoing cardiopulmonary bypass (CPB) is a significant clinical problem and has long been recognized by health professionals [6].

Postoperative pulmonary complications are highly prevalent, ranging from 5% to 20%, primarily due to perioperative factors and the intimate anatomical and functional relationship between the heart and lungs. These complications have been consistently associated with elevated postoperative morbidity and mortality rates [7, 8]. The etiology of pulmonary complications stems from a multifactorial process. Surgical factors like the use of cardiopulmonary bypass (CPB), anesthesia, surgical duration, mechanical ventilation duration, pleural opening, phrenic nerve alteration, use of mammary artery in myocardial revascularization surgery, sternal wound pain, and surgical drains lead to decreased functional residual capacity and increased intrapulmonary shunting. Furthermore, preoperative patient-related factors such as pre-existing lung diseases, smoking, advanced age, poor nutritional health, among others, predispose to complications [9].

Certain measures are employed during the postoperative period of cardiac surgeries to minimize pulmonary complications, including proper analgesia, oxygen therapy, and physiotherapy. Physiotherapy employs techniques such as deep breathing stimulation, cough stimulation, use of incentive spirometers, mobilization, and ambulation. However, at times these approaches fall short, and additional methods like Non-Invasive Ventilation (NIV) are employed [9].

Non-Invasive Positive Pressure Ventilation (NPPV) has been used to expedite pulmonary function recovery as well as to prevent and treat postoperative pulmonary complications [10]. NIV provides support for spontaneous ventilation. Its prophylactic use aims to reduce the incidence of endotracheal intubation, length of hospital stay, and prevent pulmonary complications. Non-invasive ventilation as a therapeutic resource was effective toward improving functionality; however, non-invasive ventilation did not influence the intensive care unit or hospitalization times of the studied cardiac patients [11]. Application of noninvasive ventilation (NIV), using face or nose masks has reduced the necessity of endotracheal intubation. It has been recognized that BiPAP/CPAP can prevent atelectasis and postoperative pneumonia, it also has beneficial effects in postoperative phase of cardiac surgery to prevent other pulmonary complications [12].

Postoperative pulmonary complications are the most common due to perioperative factors and the close anatomical and functional relationship between the heart and lungs. A prevalence of these complications ranging from 5% to 20% has been documented, and they are associated with increased postoperative morbidity and mortality [13].

Non-Invasive Ventilation (NIV) entails providing ventilatory support without utilizing an invasive artificial airway (endotracheal tube or tracheostomy tube). NIV aids breathing in patients with various conditions such as cardiogenic pulmonary edema, exacerbations of chronic obstructive pulmonary disease, and closed chest trauma. NIV improves gas exchange, assists in breathing, and reduces the need for intubation with positive pressure support. NIV diminishes the risk of ventilator-associated complications due to its non-invasive nature. As a result, NIV has been adopted to prevent complications in postoperative patients [10]. The use of NIV can be an important factor in patients undergoing cardiac surgery as part of a rehabilitation program and can also be a factor in reducing hospital stay, requiring larger studies to verify [14].

Continuous Positive Airway Pressure (CPAP) and Bilevel Positive Airway Pressure (BiPAP) are the most common methods of NIV^8^. Bilevel can be a successful tool for preventing the adverse effects of postoperative pulmonary complications after cardiac surgery [8].

Hence, in order to integrate these themes and provide further insight into the decision-making process for the optimal NIV modality in cardiac surgery patients, the objective of this research project is to conduct a randomized clinical trial and assess the efficacy of NIV in its CPAP and BiPAP modalities in comparison to conventional physiotherapy in terms of pulmonary function and clinical outcomes among patients undergoing cardiac surgery at a chosen hospital in Campina Grande, Paraíba, Brazil.

## Objectives

### Primary objective

Conducting a randomized clinical trial to assess the effectiveness of NIV in its CPAP and BiPAP modalities compared to conventional physiotherapy, in terms of pulmonary function and clinical outcomes among patients undergoing cardiac surgery at a selected hospital in Campina Grande, Paraíba, Brazil.

### Secondary objective

Describing the profile of cardiac patients undergoing cardiac surgery; outlining the patient profile concerning spirometry, Timed Up and Go (TUG) test, quality of life, The Global Perception of Change, vital signs, Functional Independence Measure (MIF), gasometric parameters, and pulmonary complications.

## Material and methods

### Type of study and location of research

The study protocol was conducted following the Standard Protocol Items: Recommendations for Interventional Trials (SPIRIT) checklist. This randomized, controlled, double-blind, parallel, three-arm clinical trial will take place at the João XXIII hospital, renowned for its expertise in cardiology and particularly cardiac surgery, serving a significant portion of the state and neighboring states. The hospital carries out an average of 8 cardiac surgeries weekly. The assessment and intervention will occur in both the ICU and the general ward.

The sample size was calculated using a 95% confidence level and 80% power, while considering the outcome of pulmonary function as a reference [13, 15]. Using the calculator on the openepi.com website, we arrived at a sample size of 63 patients. Recruitment will encompass both men and women aged 18 and above who have undergone cardiac surgery. Participants will be recruited through both spontaneous demand within the service and active search within the department’s records. The sampling method will be non-probabilistic convenience sampling.

### Inclusion criteria

Patients who have undergone cardiac surgery, aged 18 years or older, and are hemodynamically stable (controlled blood pressure, normal heart rate, conscious, oriented, and cooperative), with no medical restrictions for undergoing the treatment.

### Exclusion criteria

- Presence of uncontrolled cardiac arrhythmias.
- Pre-existing neuromuscular disease.
- Vestibular symptoms.
- Orthopedic/musculoskeletal restrictions.
- Unstable angina.
- Stage 3 hypertension (resting systolic blood pressure ≥ 180 mmHg and/or diastolic blood pressure ≥ 110 mmHg).
- Resting heart rate > 120 bpm.
- Systemic hypotension with clinical impact (resting systolic blood pressure ≤ 90 mmHg and/or diastolic blood pressure ≤ 60 mmHg).
- Uncontrolled arrhythmias (Examples: complete atrioventricular block, second-degree type 2 atrioventricular block, atrial fibrillation, sustained ventricular tachycardia).
- Aortic dissection.

### Post-randomization exclusion criteria

- Undergoing hospital-based treatment contraindicating study participation (e.g., pacemaker implantation, pneumothorax requiring thoracic drainage).
- Death or other decompensations unrelated to the research.
- Return to the ICU.

### Non-adherence to intervention criteria

- Patient failing to complete 3 or more consecutive sessions.
- Patient voluntarily withdrawing from the study.
- Patient experiencing cardiac arrhythmia (atrial fibrillation, bradycardia) in two sessions despite medication and rest breaks.

### Non-retention criteria

- Patient not completing any of the follow-up evaluations.

### Professional eligibility criteria

- Hospital team physiotherapists.
- Physiotherapy students with prior training in the research protocol.

The sample will be randomized using the website www.randomization.com into three groups: participants will be divided into three groups (CPAP group - CPAP + usual physiotherapy care, BiPAP group - BiPAP + usual physiotherapy care, and control group - usual physiotherapy care), in a 1:1:1 allocation ratio for superiority. The groups will be coded, and the allocation will be concealed in consecutively numbered sealed and opaque envelopes. This study will involve three investigators: the first will be responsible for assessments; the second will manage the protocol application; and the third will oversee randomization.

This study protocol has received approval from the research ethics committee of Universidade Estadual da Paraíba, Brazil (CAAE: 65600722.1.0000.5187), and has been submitted to Clinical Trials (NCT05966337). Patient autonomy and anonymity will be respected, ensuring the privacy of personal data in accordance with Resolution No. 510/16 of the National Health Council and the Declaration of Helsinki. Before enrolling in the study, all patients will provide informed consent.

The study protocol was conducted following the Standard Protocol Items: Recommendations for Interventional Trials (SPIRIT) checklist [16].

### Interventions

The intervention will initially take place in the specific ward that receives cardiac surgery patients. Participant recruitment will begin upon the patient’s admission the day before the surgery, during which they will undergo a pre-operative assessment based on the study criteria.

Physiotherapists and physiotherapy students will receive training regarding the research protocol through two in-person sessions comprising demonstrations and didactic materials. The same therapists will be responsible for both the intervention and control groups.

Patients included in the study will undergo evaluations at two time points: pre-operatively (initial assessment) and post-operatively (five days after the pre-operative assessment).

Following the initial assessment, the patient will undergo cardiac surgery the next day and will be transferred to the ICU after the procedure. The protocol will be initiated in the ICU for all three groups of the research.

#### Control group (Usual care)

Patients undergoing standard physiotherapy treatment, consisting of a kinesiotherapy protocol. Patients will receive treatment twice a day, alternating between the treatment initiated in the ICU and progressing to the general ward. Specifically, the treatment plan is divided by days and environments, taking into consideration the general characteristic that patients are discharged from the ICU on the 2nd day of the post-operative period, and Day 1 represents the first day of post-operation:

### Day 1 (ICU)

• Diaphragmatic breathing exercises – 1 set of 10 repetitions

• Cough stimulation

• Diaphragmatic breathing exercises associated with upper limbs – Shoulder flexion/extension 2 sets of 10 reps up to 90 degrees

• Shoulder abduction 1 set of 10 reps (avoiding pain)

• Diaphragmatic breathing exercises associated with lower limbs – Thigh flexion 1 set of 10 reps

• Dorsiflexion/plantar flexion of foot 1 set of 10 reps

### Day 2 (ICU)

• Diaphragmatic breathing – 1 set of 10 reps

• Cough stimulation

• Breathing exercises associated with upper limbs – Shoulder flexion 2 sets of 10 reps

• Breathing exercises associated with lower limbs – Thigh flexion 1 set of 10 reps

• Dorsiflexion/plantar flexion of foot 1 set of 10 reps

• Cycle ergometer for 3 minutes

• Breathing exercise with incentive spirometer (SMI) 1 set of 10 reps

### Day 3 (General Ward)

• Diaphragmatic breathing – 1 set of 10 reps

• Cough stimulation

• Breathing exercises associated with upper limbs – Shoulder flexion 2 sets of 10 reps

• Breathing exercises associated with lower limbs – Thigh flexion 1 set of 10 reps

• Dorsiflexion/plantar flexion of foot 1 set of 10 reps

• Ambulation for 5 minutes

### Day 4 (General Ward)

• Diaphragmatic breathing – 1 set of 10 reps

• Cough stimulation

• Ambulation for 10 minutes

### Day 5 (General Ward)

• Diaphragmatic breathing – 1 set of 10 reps

• Cough stimulation

• Ambulation for 15 minutes

#### Intervention group 1 (CPAP)

Patients in this group will receive the same care as the control group, with the addition of Non-Invasive Ventilation (NIV) using nasal Continuous Positive Airway Pressure (CPAP) at 10 cmH2O pressure for 1 hour. The CPAP device from the brand RESMED will be used. This intervention will be administered for a duration of 5 days throughout the patient’s hospitalization, both in the ICU and general ward. The frequency of sessions will be twice (2 times) per day, in the morning and afternoon.

Flexibility in scheduling the intervention sessions is planned, considering that hospital environments often involve examinations and other procedures that might impact the initially scheduled protocol times. After the fifth day, the patient will undergo a reassessment using the same measurement instruments as before.

#### Intervention group 2 (BIPAP)

Patients in this group will receive the same care as the control group, with the addition of Non-Invasive Ventilation (NIV) using nasal Bi-Level Positive Airway Pressure (BIPAP) with an Inspiratory Positive Airway Pressure (IPAP) of 13 cmH2O and an Expiratory Positive Airway Pressure (EPAP) of 8 cmH2O for 1 hour. The BIPAP device from the brand RESMED will be used. This intervention will be administered for a duration of 5 days throughout the patient’s hospitalization, both in the ICU and general ward. The frequency of sessions will be twice (2 times) per day, in the morning and afternoon.

Similar to the CPAP group, flexibility in scheduling the intervention sessions is planned to accommodate the dynamic nature of hospital settings. After the fifth day, the patient will undergo a reassessment using the same measurement instruments as before.

### Data collection

**Fig 1:**
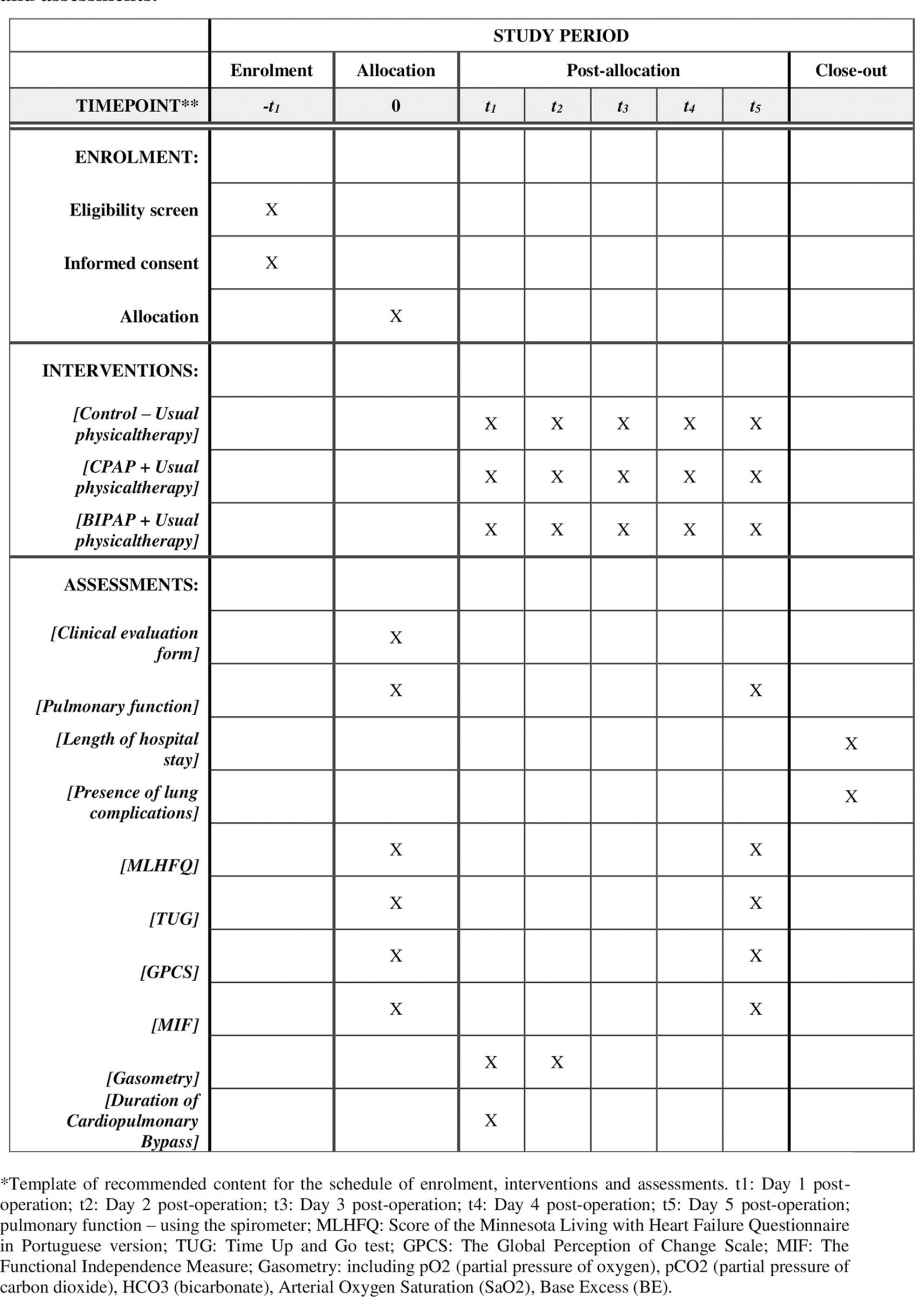
The recommendations for interventional trials (SPIRIT) schedule of enrollment, interventions, and assessments.

The tests and questionnaires used for these assessments are listed along with their outcome variables in Fig 1.

#### Clinical evaluation form

After signing the informed consent form, patients will be assessed using an evaluation form. In this form, on the first day, the assessment will encompass both anamnesis (patient history) and physical examination, with a focus on the patient’s cardiac history. This will be followed by measurements specific to the protocol, including chest X-ray, quality of life questionnaire, Timed Up and Go (TUG) test, and spirometry. The assessment may be divided into two stages if there are other demands on the patient during their hospitalization.

#### Pulmonary function

All participants will undergo an evaluation by a CONTEC model spirometer (SPB80b, CHINA) to verify lung volumes and capacities. At least three tests will be performed, with a variation of less than 5% and the highest value obtained in one of the tests will be compared with the predicted values of pulmonary function parameters for the Brazilian population. FEV1 and FVC will be assessed. The predicted values will be calculated using the reference values [17].

#### Length of hospital stay

Absolute number of days of hospitalization up to 2 weeks after the date of randomization.

#### Quality of life

The Portuguese version of the MLHFQ will be used to assess the patient’s quality of life. The questionnaire provides a final score as a metric, and the change in score over the treatment period will be evaluated. The MLHFQ score is a measure of health-related quality of life in patients with heart failure. The higher the score, the greater the negative impact of heart failure on the patient’s quality of life. A score of 0 indicates that the patient has no limitations or problems related to heart failure, while a score of 105 indicates a maximum limitation or problem in all areas assessed by the questionnaire [18].

#### Funcional capacity

The TUG test will be performed to assess functional capacity. The patient will start from a sitting position, walk quickly for 3 meters, turn 180 degrees, and return to the chair. The time taken, measured from standing up to completely leaning the trunk against the chair, will be recorded. Time values below 10 seconds indicate maximum functionality, 10 to 19 seconds indicate functional independence, 20 seconds and above indicate some functional limitation, and 30 seconds and above indicate significant functional limitation [19].

#### Perception of change

The GPCS is a self-report tool that assesses an individual’s perception of change in a specific domain. It uses Likert-scale items ranging from "a very great deal worse/less" to "a very great deal better/more" to capture subjective information about perceived change [20].

#### Functional Independence Measure (MIF)

The MIF questionnaire will be administered preoperatively and on the sixth day to assess functional independence. Adaptado do questionário de Borges, 2006 [21].

#### Gasometric data

Includes pO2 (partial pressure of oxygen), pCO2 (partial pressure of carbon dioxide), HCO3 (bicarbonate), Arterial Oxygen Saturation (SaO2), and Base Excess (BE). These measurements will be collected on Day 1 and Day 2, following the ICU routine and based on the patient’s clinical needs.

#### Presence of lung complications

Assessment of the occurrence or absence of any complications related to the lungs during the hospitalization period cause, whichever occurs first, measured up to 2 weeks. The time frame from the date of randomization to the date of the first documented progression or the date of death from any cause, whichever occurs first, measured up to 2 weeks after the date of hospital discharge.

#### Duration of cardiopulmonary bypass

The duration of cardiopulmonary bypass during the cardiac surgery.

#### Other variables

Will be collected through the analysis of the patient’s medical records, including the type of surgery, personal history, pre-operative ejection fraction, BMI, gender, and age.

*Adherence*. During the assessment and daily sessions, therapists will be instructed to raise awareness, provide guidance, and explain the purpose of the research to ensure maximum adherence to the protocol. Adherence assessment will involve direct communication between the researcher and the participating physiotherapists and students, facilitated through a dedicated messaging app group created for this purpose. Monitoring adherence will also include regular visits by the researcher during the application of the protocol treatment.

*Concomitant care*. Patients requiring extended ICU stays with the use of more advanced devices for prolonged non-invasive ventilation or even the return to invasive mechanical ventilation during the study period will be considered non-adherent from that point, and thus an intention-to-treat analysis will be employed.

*Risks and adverse effects*. Throughout the treatment, there are minimal risks of cardiac arrhythmias, decreased oxygen saturation levels, shortness of breath, bronchospasm, dizziness, syncope, hypotension or hypertension, altered heart rate, or chest pain. Throughout the entire duration, patients will be continuously monitored for vital signs.

#### Intervention protocol

The following training protocols will be applied.

*Control group (Usual physicaltherapy)*. Patients undergo physiotherapy treatment twice daily, transitioning from the ICU to the ward. In detail, the daily sessions are divided based on the days and settings, taking into account that patients are generally discharged from the ICU on Day 2 post-operation and that Day 1 represents the first day of post-operative care:

### DAY 1 (ICU)

Diaphragmatic breathing exercises – 1 set of 10 repetitions

Cough stimulation

Diaphragmatic breathing exercises combined with upper limb movements – Shoulder flexion/extension 2 sets of 10 repetitions up to 90 degrees

Diaphragmatic breathing exercises combined with lower limb movements – Thigh flexion 1 set of 10 repetitions, Dorsiflexion/plantar flexion 1 set of 10 repetitions

### DAY 2 (ICU)

Diaphragmatic breathing – 1 set of 10 repetitions

Cough stimulation

Diaphragmatic breathing exercises combined with upper limb movements – Shoulder flexion 2 sets of 10 repetitions

Diaphragmatic breathing exercises combined with lower limb movements – Thigh flexion 1 set of 10 repetitions, Dorsiflexion/plantar flexion 1 set of 10 repetitions

Cycle ergometer for 3 minutes

Breathing exercise with SMI (Strength Measuring Instruments) – 1 set of 10 repetitions

### DAY 3 (WARD)

Diaphragmatic breathing – 1 set of 10 repetitions

Cough stimulation

Diaphragmatic breathing exercises combined with upper limb movements – Shoulder flexion 2 sets of 10 repetitions

Diaphragmatic breathing exercises combined with lower limb movements – Thigh flexion 1 set of 10 repetitions, Dorsiflexion/plantar flexion 1 set of 10 repetitions

Ambulation (walking) for 5 minutes

### DAY 4 (WARD)

Diaphragmatic breathing – 1 set of 10 repetitions

Cough stimulation

Ambulation (walking) for 10 minutes

### DAY 5 (WARD)

Diaphragmatic breathing – 1 set of 10 repetitions

Cough stimulation

Ambulation (walking) for 15 minutes

*CPAP + Usual physicaltherapy*. They will be submitted to the same care as the control group, adding NIV with nasal CPAP 10cmH2O for 1 hour using device and brand approved by ANVISA, during the 5 days of hospitalization, both in the ICU and in the ward. The frequency of the sessions will be two (2) per day, in the morning and in the afternoon. Flexibility of time for carrying out the procedure is also planned, since in a hospital environment the patient can often undergo exams, other behaviors that may make it difficult to apply the protocol at the initially scheduled time.

*BIPAP + Usual physicaltherapy*. They will undergo the same care as the control group, adding NIV with nasal BIPAP with IPAP of 13cmH2O and EPAP 8 cmH2O for 1 hour, using equipment and brand approved by ANVISA, during the 5 days of hospitalization, both in the ICU and in the ward. The frequency of the sessions will be two (2) per day, in the morning and in the afternoon. Flexibility of time for carrying out the procedure is also planned, since in a hospital environment the patient can often undergo exams, other behaviors that may make it difficult to apply the protocol at the initially scheduled time. After the fifth day, the patient will be reassessed with the same instruments reported.

### Statistical analysis

The data will be analyzed using IBM SPSS Statistics Base 25.0 software for Windows. The normality of variables will be assessed using the Shapiro-Wilk test. The paired Student’s t-test will be utilized to compare data before and after the intervention. Linear Mixed Effects Models will be used for longitudinal data analysis. The effect of NIV (CPAP or BIPAP) combined with usual physiotherapy over time will be compared between groups using generalized linear mixed models (GLMMs), they can account for the correlation between repeated measurements within the same individual, which is important in longitudinal studies. The intention-to-treat (ITT) approach will be used to handle potential missing data. Time dependence is a key consideration in data analysis, and autoregression, particularly Integer Auto Regressive (INAR) models, is a valuable method for modeling this. However, selecting the appropriate autoregressive model is essential for gaining insights into the temporal dynamics of medical outcomes. Fixed effects models as a choice for addressing endogeneity. We use technique Bayesian model averaging to select relevant variables while controlling for overfitting. To ensure analysis accuracy, parametric model assumptions, such as homoscedasticity and residual normality, will be checked. Moreover, various models will be considered to accommodate specific data characteristics, such as skewness and zero-inflation/overdispersion.

Effect sizes will be calculated using Cohen’s d. Continuous variables will be reported as mean ± standard deviation (SD) along with a 95% confidence interval (CI), while categorical variables will be presented as absolute frequencies and percentages. The significance level will be set at 5% for all analyses (p ≤ 0.05).

#### Harms

A specific form for adverse effects will be developed, provided in supplementary material, and distributed to the professionals and students implementing the protocol. Adverse events will be recorded and sent weekly to the data monitoring committee.

#### Audit

The data monitoring committee will conduct planned technical visits to the hospital during the protocol’s implementation to verify procedures.

#### Protocol modifications

No modifications will be made to the protocol after its publication in clinical trials database.

#### Consent or assent

Every participant must sign an informed consent form (Appendix 1) on the day of hospital admission. A student or professional conducting the initial assessment will request the patient’s signature.

#### Confidentiality

Under no circumstances will personal information be disclosed, and collected data will be kept confidential and published only in an impersonal manner, ensuring data confidentiality in specific scientific journals.

#### Complementary and post-trial treatments

In case of any harm or adverse effect resulting from the intervention, the patient can seek compensation for possible damage.

## Supporting information

S1 Checklist SPIRIT 2013 checklist: Recommended items to address in a clinical trial protocol and related documents*. (DOC)

S1 Fig (DOC)

S1 File (DOC)

S2 File (DOC)
