# Supplementary material for: Effects of Non-Invasive Ventilation with different modalities in patients undergoing heart surgery: Protocol for a randomized controlled clinical trial
Source: PLoS One. 2024 Jun 18;19(6):e0304569. doi: 10.1371/journal.pone.0304569 (PMC11185470; doi:10.1371/journal.pone.0304569)
Supplement: S1 Fig — (DOC) [file pone.0304569.s002.doc]

**Figure 1**.**Template of recommended content for the schedule of enrolment, interventions, and assessments.***

|  | **STUDY PERIOD** | | | | | | | |
| --- | --- | --- | --- | --- | --- | --- | --- | --- |
|  | **Enrolment** | **Allocation** | **Post-allocation** | | | | | **Close-out** |
| **TIMEPOINT**** | ***-t_1_*** | **0** | ***t_1_*** | ***t_2_*** | ***t_3_*** | ***t_4_*** | ***t_5_*** |  |
| **ENROLMENT:** |  |  |  |  |  |  |  |  |
| **Eligibility screen** | X |  |  |  |  |  |  |  |
| **Informed consent** | X |  |  |  |  |  |  |  |
| **Allocation** |  | X |  |  |  |  |  |  |
| **INTERVENTIONS:** |  |  |  |  |  |  |  |  |
| ***[Control – Usual physicaltherapy]*** |  |  | X | X | X | X | X |  |
| ***[CPAP + Usual physicaltherapy]*** |  |  | X | X | X | X | X |  |
| ***[BIPAP + Usual physicaltherapy]*** |  |  | X | X | X | X | X |  |
| **ASSESSMENTS:** |  |  |  |  |  |  |  |  |
| ***[Clinical evaluation form]*** |  | X |  |  |  |  |  |  |
| ***[Pulmonary function]*** |  | X |  |  |  |  | X |  |
| ***[******Length of hospital stay]*** |  |  |  |  |  |  |  | X |
| ***[******Presence of lung complications]*** |  |  |  |  |  |  |  | X |
| ***[******MLHFQ]*** |  | X |  |  |  |  | X |  |
| ***[TUG]*** |  | X |  |  |  |  | X |  |
| ***[GPCS]*** |  | X |  |  |  |  | X |  |
| ***[******MIF]*** |  | X |  |  |  |  | X |  |
| ***[******Gasometry]*** |  |  | X | X |  |  |  |  |
| ***[******Duration of Cardiopulmonary Bypass]*** |  |  | X |  |  |  |  |  |

*Template of recommended content for the schedule of enrolment, interventions and assessments. t1: Day 1 post-operation; t2: Day 2 post-operation; t3: Day 3 post-operation; t4: Day 4 post-operation; t5: Day 5 post-operation; pulmonary function – using the spirometer; MLHFQ: Score of the Minnesota Living with Heart Failure Questionnaire in Portuguese version; TUG: Time Up and Go test; GPCS: The Global Perception of Change Scale; MIF: The Functional Independence Measure; Gasometry: including pO2 (partial pressure of oxygen), pCO2 (partial pressure of carbon dioxide), HCO3 (bicarbonate), Arterial Oxygen Saturation (SaO2), Base Excess (BE).
