# Supplementary material for: Effects of Non-Invasive Ventilation with different modalities in patients undergoing heart surgery: Protocol for a randomized controlled clinical trial
Source: PLoS One. 2024 Jun 18;19(6):e0304569. doi: 10.1371/journal.pone.0304569 (PMC11185470; doi:10.1371/journal.pone.0304569)
Supplement: S1 File — (DOC) [file pone.0304569.s003.doc]

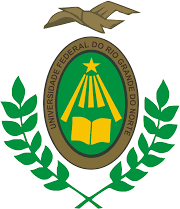


# UNIVERSIDADE FEDERAL DO RIO GRANDE DO NORTE ´

# CENTRO DE CIÊNCIAS DA SAÚDE

**PROGRAMA DE PÓS-GRADUAÇÃO EM FISIOTERAPIA**

**DISCIPLINA: PROJETOS DE ENSAIO CLÍNICO EM FISIOTERAPIA E DESENVOLVIMENTO DE PROTOCOLOS**

**DOCENTE: DRA PATRÍCIA ANGÉLICA DE MIRANDA SILVA NOGUEIRA**

**DISCENTE: EDER RODRIGUES ARAÚJO**

**EFEITOS DA VENTILAÇÃO NÃO-INVASIVA COM DIFERENTES MODALIDADES EM PACIENTES SUBMETIDOS A CIRURGIA CARDÍACA: UM ENSAIO CLÍNICO RANDOMIZADO CONTROLADO**

NATAL - RN

2022

# INFORMAÇÕES GERAIS

# Este protocolo é parte da tese de doutorado de Eder Rodrigues Araújo, sob orientação da prof Dra Patrícia Angélica de Miranda Silva Nogueira e será registrado no Registro Brasileiro de Ensaios Clínicos (REBEC). Trata-se da versão nº 1 – 22/11/2022.

# Financiamento: Este estudo será financiado em parte pela Coordenação de Aperfeiçoamento de Pessoal de Nível Superior - Brasil (CAPES) - Código Financeiro 001. O estudo contará ainda com parceria com o núcleo de tecnologias em saúde (NUTES-UEPB) para financiamento de dispositivos vestíveis de monitoramento remoto referentes à pesquisa.

# Funções e responsabilidades:

# Eder Rodrigues Araújo, UFRN, doutorando, eder.rodrigues.araujo@gmail.com autor principal do protocolo, participará do treinamento dos fisioterapeutas para aplicação do protocolo e das análises posteriores.

# Patrícia Angélica de Miranda Silva Nogueira, UFRN, professora doutora, idpa02@hotmail.com, orientadora, coautora do protocolo, participará da supervisão técnica da redação do protocolo, das análises e discussões do estudo e da coordenação do comitê de monitoramento de dados.

# NUTES/UEPB: Instituição de tecnologia da Universidade Estadual da Paraíba que tem parceria com o laboratório de medidas e avaliação em saúde da UFRN. Participarão com a aquisição e disponibilização de smartwatches para monitoramento remoto de sinais vitais dos pacientes.

# RESUMO

# O procedimento cirúrgico torácico acarreta redução da força muscular respiratória. Para restabelecê-la, algumas estratégias devem ser utilizadas. A fisioterapia utiliza os recursos e técnicas, como estimulação da respiração profunda, estimulo à tosse, uso de espirômetros de incentivo, mobilização e deambulação. No entanto, às vezes esses recursos e técnicas não são suficientes, e recursos adicionais, como o uso de VNI são empregados^19^. A ventilação não invasiva com pressão positiva (NPPV) tem sido usada para acelerar a recuperação da função pulmonar, bem como prevenir e tratar complicações pulmonares pós-operatórias ^16^. A VNI reduz o risco de complicações associadas ao ventilador devido às suas características não invasivas. Assim, a VNI tem sido adotada para prevenir complicações após a extubação em pacientes pós-operatórios^18^. O objetivo deste trabalho é realizar um ensaio clínico randomizado e verificar a eficácia da VNI comparada à fisioterapia convencional no âmbito do índice de complicações pulmonares em pacientes submetidos a cirurgia cardíaca em hospital escolhido na cidade de Campina Grande, Paraíba Trata-se de um ensaio clínico randomizado controlado, duplo cego (paciente, analista) que propõe a intervenção com utilização no grupo BIPAP da VNI modalidade BIPAP em um grupo com cuidados habituais de fisioterapia e no grupo CPAP da VNI modalidade CPAP em um grupo com cuidados habituais de fisioterapia e o grupo controle com apenas cuidados habituais de fisioterapia. Os desfechos observados serão: avaliação entre as modalidades de VNI, espirometria, capacidade pulmonar e sinais vitais. Desfechos secundários incluídos são grau de satisfação pela escala de perceção global de mudança, e MIF – medida de independência funcional e parâmetros gasométricos. Espera-se que os resutados dessa pesquisa possam contribuir para verificação da VNI no paciente cardiopata cirúrgico na prática clínica.

# INTRODUÇÃO

Nos Estados Unidos, mais de 2.200 americanos morrem todos os dias de causas cardiovasculares, e pessoas com mais de 65 anos são responsáveis por quase 80% dessas mortes. A *American Heart Association* refere que os adultos mais velhos também são responsáveis por quase 75% das doenças cardiovasculares. A carga nacional de morbidade e mortalidade causada por eventos cardiovasculares permanece alta, e existe uma busca por medidas preventivas eficientes e eficazes.^5^

Em pacientes com doenças cardiovasculares, incluindo doença valvar, as diretrizes atuais aconselham considerar a capacidade de exercício para diagnóstico e planejamento de tratamento.^6,7^ Além disso, A doença arterial coronariana é a principal causa de óbito no mundo e os pacientes submetidos à cirurgia de revascularização miocárdica (CRVM) constituem o grupo de maior risco.^8^

O aumento constante de pacientes com cardiopatia denota a necessidade de gerar novos dispositivos adaptáveis para a realidade desses pacientes. Medidas de avanços são necessários para promover melhores serviços a este grupo de risco. ^9,10^

As complicações respiratórias continuam sendo uma das principais causas de morbidade em pacientes de cirurgia cardíaca^17^. A etiologia das complicações pulmonares resulta de um processo multifatorial. Fatores cirúrgicos como uso de circulação extracorpórea (CEC), anestesia, tempo de cirurgia, tempo de ventilação mecânica, abertura pleural, alteração do nervo frênico, uso da artéria mamária em cirurgia de revascularização do miocárdio, dor na ferida operatória esternal e nos drenos cirúrgicos levar à diminuição da capacidade residual funcional e aumento do shunt intrapulmonar. Além disso, fatores pré-operatórios relacionados ao paciente, como doenças pulmonares preexistentes, tabagismo, idade avançada, má saúde nutricional, entre outros, predispõem a complicações. ^19^

Certas medidas são utilizadas durante o PO de cirurgias cardíacas, na tentativa de minimizar as complicações pulmonares, como analgesia adequada, oxigenoterapia e fisioterapia. A fisioterapia utiliza os recursos e técnicas, como estimulação da respiração profunda, estimulo à tosse, uso de espirômetros de incentivo, mobilização e deambulação. No entanto, às vezes esses recursos e técnicas não são suficientes, e recursos adicionais, como o uso de VNI são empregados. ^19^

A ventilação não invasiva com pressão positiva (NPPV) tem sido usada para acelerar a recuperação da função pulmonar, bem como prevenir e tratar complicações pulmonares pós-operatórias. ^16^

A VNI é um suporte para ventilação espontânea. Seu uso como medida profilática visa reduzir a incidência de intubação endotraqueal, tempo de internação e prevenir complicações pulmonares. No entanto, mesmo com ensaios clínicos randomizados (ECRs) e revisões sistemáticas, não há consenso na literatura quanto ao seu uso como medida profilática após cirurgia cardíaca

As complicações pulmonares pós-operatórias são as mais comuns devido aos fatores perioperatórios, bem como à estreita relação anatômica e funcional entre o coração e os pulmões. Uma prevalência dessas complicações de 5% a 20% foi documentada e a associação com aumento da morbidade e mortalidade pós-operatórias. ^18^

A ventilação não invasiva (VNI) denota a administração de suporte ventilatório sem o uso de uma via aérea artificial invasiva (tubo endotraqueal ou cânula de traqueostomia). A VNI apoia a respiração em pacientes com várias condições, como edema pulmonar cardiogênico, exacerbações de doença pulmonar obstrutiva crônica e aqueles que sofreram trauma fechado no tórax. A VNI melhora as trocas gasosas, auxilia a respiração e reduz a necessidade de intubação com suporte de pressão positiva. A VNI reduz o risco de complicações associadas ao ventilador devido às suas características não invasivas. Assim, a VNI tem sido adotada para prevenir complicações após a extubação em pacientes pós-operatórios. ^18^

A pressão positiva contínua nas vias aéreas (CPAP) e a pressão positiva nas vias aéreas em dois níveis (BIPAP) são os métodos mais comuns de VNI. ^20^

Portanto, com o intuito de integrar essas temáticas, e estabelecer mais subsídio para decisão da melhor modalidade de VNI em pacientes de cirurgia cardíaca, o objetivo desse projeto de pesquisa é realizar o ensaio clínico randomizado e verificar a eficácia da VNI em suas modalidades CPAP e BIPAP comparada à fisioterapia convencional no âmbito do índice de complicações pulmonares, função pulmonar e desfechos clínicos em pacientes submetidos a cirurgia cardíaca em hospital escolhido na cidade de Campina Grande, Paraíba.

# OBJETIVO

Realizar ensaio clínico randomizado verificar a eficácia da VNI em suas modalidades CPAP e BIPAP comparada à fisioterapia convencional no âmbito do índice de complicações pulmonares, função pulmonar e desfechos clínicos em pacientes submetidos a cirurgia cardíaca em hospital escolhido na cidade de Campina Grande, Paraíba.

# Objetivos Específicos

- Desenvolver uma plataforma de integração de todos os dados clínicos vindos dos pacientes submetidos a cirurgia cardíaca;
- Descrever perfil dos pacientes cardiopatas submetidos a cirurgia cardíaca;
- Facilitar o trabalho realizado pelos profissionais envolvidos nos cuidados com pacientes cardiopatas submetidos a cirurgia;
- Traçar perfil dos pacientes em relação a espirometria, teste de caminhada de 6 minutos, satisfação, sinais vitais, MIF e parâmetros gasométricos.

# Desenho do estudo

# O protocolo de estudo foi conduzido seguindo o checklist Standard Protocol Items: Recommendations for Interventional Trials (SPIRIT). Trata-se de um ensaio clínico randomizado controlado, duplo cego, onde foram cegados participantes e avaliadores de desfechos, e unicêntrico. O estudo será submetido ao comitê de ética da Universidade Estadual da Paraíba (UEPB) e realizado de acordo com a Declaração de Helsinque. Todos os participantes devem fornecer consentimento informado por escrito antes da participação, e o estudo será registrado na plataforma REBEC. Todos os participantes devem fornecer consentimento informado por escrito antes da participação. Os participantes serão divididos em tres grupos (*grupo cpap* – CPAP + cuidados habituais de fisioterapia, *grupo bipap –* BIPAP + cuidados habituais de fisioterapia e *controle* – cuidados habituais de fisioterapia), na razão de alocação 1:1 e de superioridade.

# METODOS

**Participantes, intervenções e desfechos**

# Local do estudo

O projeto será realizado no Hospital João XXIII na cidade de Campina Grande, Paraíba, Brasil. Trata-se de um hospital privado conveniado a rede SUS referência em cardiologia e especialmente em cirurgia cardíaca para boa parte da região do estado e de estados circunvizinhos. Apresenta em seu programa semanal uma média de 8 cirurgias cardíacas. **A avaliação e intervenção ocorrerão na UTI e enfermaria.** O Termo de Autorização Institucional para a realização legal da pesquisa já encontra-se assinado (APÊNDICE A).

**Critério de elegibilidade**

**Critérios de inclusão**: Pacientes submetidos a cirurgia cardíaca, com idade acima de 18 anos, estáveis do ponto de vista hemodinâmico (pressão arterial controlada, normocárdico – consciente e orientado e cooperativo) sem restrições médicas para realização do tratamento.

**Critérios de exclusão**: Presença de arritmias cardíacas não controladas, doença neuromuscular prévia, labirintite e alguma restrição ortopédica/músculo esquelética. Angina instável, HAS estágio 3 (PAS ≥ 180 mmHg e/ou PAD ≥ 110 mmHg no repouso), FC > 120 bpm no repouso, hipotensão arterial sistêmica com repercussão clínica (PAS ≤ 90 mmHg e/ou PAD ≤ 60 mmHg), arritmias descontroladas (Exemplos: bloqueio átrio-ventricular total, bloqueio átrio-ventricular do 2º grau tipo 2, fibrilação atrial, taquicardia ventricular sustentada), dissecção de aorta.

**Critérios de exclusão pós-randomização**: No caso de realização de algum tratamento no âmbito hospitalar que contraindique a participação no estudo (implante de marcapasso, pneumotórax com necessidade de drenagem torácica por ex), óbito ou outras descompensações não relacionadas à pesquisa e retorno à UTI.

**Critérios de não aderência à intervenção**: Paciente que não realize 3 ou mais sessões seguidas, que desistiu de participar do estudo e que apresente alguma arritmia cardíaca (fibrilação atrial, bradicardia) por duas sessões mesmo após medicação utilizada para conter e pausa para descanso.

**Critérios de não-retenção: Paciente que não realize uma das reavaliações.**

**Critérios de elegibilidade dos profissionais: Fisioterapeutas da equipe hospitalar e estudantes de fisioterapia com treinamento prévio do protocolo da pesquisa.**

**Intervenção**

**A intervenção ocorrerá inicialmente na enfermaria específica que recepciona os pacientes em pós-operatório de cirurgia cardíaca. O recrutamento dos participantes acontecerá a partir da internação do paciente no dia anterior da cirurgia, período no qual será submetido a avaliação pré-operatória com os critérios do estudo. Nesse primeiro dia a avaliação será composta por anamnese e exame físico, direcionando-se para histórico de distúrbios cardíacos, seguida de mensurações inerentes ao protocolo. Manovacuometria, radiografia de tórax, questionário de qualidade de vida, teste de caminhada e espirometria. A avaliação poderá ser realizada em duas etapas, caso existam outras demandas para o paciente durante sua internação hospitalar.**

**Os fisioterapeutas e estudantes de fisioterapia serão treinados em relação ao protocolo da pesquisa, perfazendo dois encontros presenciais com demonstrações e material didático. Tanto para o grupo intervenção quando o controle os terapeutas serão os mesmos.**

**O início da intervenção está previsto para Março de 2023, após aprovação em comitê de ética e pesquisa e ajustes de tratativas burocráticas com o hospital.**

**Grupo controle (Cuidados habituais): Pacientes submetidos a tratamento usual da fisioterapia, composto por protocolo de cinesioterapia. Os pacientes serão submetidos duas vezes ao dia, intercalando o tratamento já iniciado em ambiente de UTI e progredindo para a enfermaria. Detalhadamente, temos uma divisão por dias e ambientes, observando-se a característica geral do paciente ter alta da UTI no 2 dia de PO e que o dia 1 representa o primeiro dia de Pós-operatório:**

**DIA 1 (UTI):** Exercícios respiratórios diafragmáticos – 1 x 10; Estímulo à tosse; Exercícios respiratórios diafragmáticos associados a MMSS – Flexão/extensão de ombro 2 x 10 até 90 graus; Abdução de ombro 1 x 10 (retirar para evitar dor); Exercícios respiratórios diafragmáticos associados a MMII – Flexão de coxa 1 x 10; Dorsiflexão/flexão plantar 1 x 10**.**

**DIA 2 (UTI):** Respiração diafragmática – 1 x 10; Estímulo à tosse; Exercícios respiratórios associados a MMSS – Flexão de ombro 2 x 10; Exercícios respiratórios associados a MMII – Flexão de coxa 1 x 10; Dorsiflexão/flexão plantar 1 x 10; Cicloergômetro 3 min; Exercício respiratório com SMI 1x10

**DIA 3 (ENFERMARIA):** Respiração diafragmática – 1 x 10; Estímulo à tosse; Exercícios respiratórios associados a MMSS – Flexão de ombro 2 x 10; Exercícios respiratórios associados a MMII – Flexão de coxa 1 x 10; Dorsiflexão/flexão plantar 1 x 10; Deambulação 5 min.

**DIA 4 (ENFERMARIA):** Respiração diafragmática – 1 x 10; Estímulo à tosse; Deambulação 10 min.

**DIA 5 (ENFERMARIA):** Respiração diafragmática – 1 x 10; Estímulo à tosse; Deambulação 15 min.

**Grupo intervenção 1 (CPAP): Serão submetidos aos mesmos cuidados do grupo controle, adicionando-se a VNI com** CPAP nasal 10cmH2O por 1 hora **utilizando aparelho _________________marca ___________, durante os 5 dias de internação, tanto em UTI quanto na enfermaria. A frequência das sessões será de duas (2) por dia, no período da manhã e da tarde. A flexibilização de horário para realização da conduta também está prevista, uma vez que em ambiente hospitalar frequentemente o paciente pode passar por exames, outras condutas que podem dificultar a aplicação do protocolo no horário previsto inicialmente. Após o quinto dia o paciente será reavaliado com os mesmos instrumentos relatados.**

**Grupo intervenção 2 (BIPAP): Serão submetidos aos mesmos cuidados do grupo controle, adicionando-se a VNI com** BIPAP nasal com IPAP de 13cmH2O e EPAP 8 cmH2O por 1 hora, **utilizando aparelho _________________marca ___________, durante os 5 dias de internação, tanto em UTI quanto na enfermaria. A frequência das sessões será de duas (2) por dia, no período da manhã e da tarde. A flexibilização de horário para realização da conduta também está prevista, uma vez que em ambiente hospitalar frequentemente o paciente pode passar por exames, outras condutas que podem dificultar a aplicação do protocolo no horário previsto inicialmente. Após o quinto dia o paciente será reavaliado com os mesmos instrumentos relatados.**

**Aderência: Durante a avaliação e conduta nas sessões diárias, o terapeuta será instruído a realização de conscientização, orientações, e descrição do propósito da pesquisa, a fim de obter a máxima aderência ao protocolo. A avaliação da aderência será realizada com contato direto do pesquisador com os fisioterapeutas e estudantes do estudo em grupo de aplicativo de mensagem previamente formado especificamente para esse intuito. A monitorização da aderência vai também contar com visitas frequentes do pesquisador no momento da aplicação do tratamento protoocolar.**

**Cuidados concomitantes: Os pacientes que necessitem de tempo maior de internação em UTI com utilização de maiores dispositivos para permanência prolongada em ventilação não-invasiva ou até mesmo o retorno à VMI durante o período do estudo serão considerados não aderentes a partir desse período, e assim prevê-se o uso da intenção de tratar.**

**Follow-up: Previsto para um (1) mês após a alta hospitalar, realizada a reavaliação do paciente após a consulta médica de retorno da cirurgia cardíaca.**

**Desfechos: Lembrar de propriedades psicométricas, dividir em topicos desfecho e a variável.**

**Primário:**

***Capacidade pulmonar*: A espirometria verificará capacidade vital (CV), capacidade vital forçada expiratória (CVF), volume expiratório forçado no primeiro segundo (VEF1) e a relação VEF1/CVF. A métrica considerada também será vista no antes e o depois do tratamento, também verificando-se o delta. Utilizaremos equipamento espirômetro portátil Contec SP80B.**

***Tempo de permanência hospitalar*: Variável desfecho verificada pelo número de dias absoluto de internação. Desfecho importante para a verificação de custos hospitalares e complicações pós-operatórias, principalmente.**

***Complicações pulmonares*: Pacientes terão suas radiografias pré-operatórias comparadas com a radiografia torácica no dia da alta hospitalar, bem como relatos de complicações pulmonares durante a internação serão verificados, tais como presença de atelectasia, derrame pleural sem necessidade de drenagem torácica, derrame pleural com histórico de drenagem torácica. A avaliação será realizada pelo pesquisador, verificando laudos emitidos pela equipe de radiologia do hospital. A métrica considerada será a presença de complicações, verificando-se o antes e o depois.**

**Secundários:**

***Qualidade de vida*: A versão em português do *Minnesota Living with Heart Failure Questionnaire* (MLHFQ) será utilizado para avaliar a qualidade de vida do paciente ^7^. O questionário tem uma métrica final em score e será avaliado o delta na variação desse período de tratamento.**

***Capacidade funcional submáxima*: O TC6 será realizado de acordo com as diretrizes da ATS ^8^. A maior distância de caminhada de seis minutos (DTC6) foi considerada para análise e comparada com o previsto. A métrica considerada também será vista no antes e o depois do tratamento, também verificando-se o delta.** O teste de caminhada de 6 minutos será realizado baseado no protocolo inicial desenvolvido em função de *guidelines* internacionais do tema, num local apropriado cedido pelo hospital, com toda a estrutura e demarcação necessária. Após o cadastro do paciente e colocação do *smartwatch*, o pesquisador submeterá o paciente ao teste. Os dados passam a ser coletados automaticamente e colocados em plataforma já referida, com todos os sinais vitais em função do tempo do teste. Realizado no pré-operatório e no sexto dia.

*A escala de percepção global de mudança* também será aplicada no sexto dia, segundo a adaptação de Domingues, 2011.

*A medida de independência funcional (MIF)* será realizada no pré-operatório e no sexto dia, utilizando-se do questionário adaptado de Borges, 2006.

*Os dados da gasometria* serão analisados e verificados no prontuário do paciente no pós-operatório imediato, antes do paciente iniciar o protocolo.

*Outras variáveis* serão coletadas pela análise do prontuário do paciente: Tempo de circulação extracorpórea, tempo de internação hospitalar, tipo de cirurgia, antecedentes pessoais, fração de ejeção pré-operatória, complicações respiratórias durante a internação, peso, altura e IMC, sexo e idade.

**MEDIDAS DE CARACTERIZAçÂO DA AMOSTRA – MONITORAMENTO: Mensuração e monitorização de sinais vitais durante todo o tratamento.**

**Riscos e efeitos adversos: Durante o tratamento, existem** **riscos mínimos de arritmias cardíacas, quedas de níveis de saturação, dispnéia, broncoespasmo, tontura, síncope, hipotensão ou hipertensão arterial, alteração de frequência cardíaca ou dor torácica. Durante todo o período, o paciente estará monitorado em relação aos sinais vitais através do *Smartwatch Garmin Forerruner* 945 com conexão remota e medição de consumo máximo de oxigênio (VO2), saturação de oxigênio (SatO2) e frequência cardíaca (FC) com validação própria para essas variáveis. Caso ocorram essas situações acima referidas, o paciente terá o tratamento interrompido para compensação do quadro e reavaliação para retorno à pesquisa com segurança.**

**Eventos adversos: Serão reportados quaisquer eventos relacionados a distúrbio cardiovascular, utilizando-se o smartwatch. Durante todo o protocolo, o paciente será contactado e questionado sobre qualquer alteração fora do período do tratamento, por autorrelato do paciente.**

# Fluxograma (COLOCAR legenda)

|  |  | Pre-treatment  Post-allocation | | | | | | | | |
| --- | --- | --- | --- | --- | --- | --- | --- | --- | --- | --- |
|  |  | | |  | | 4 | | | |  |
| **TIMEPOINT*(days)** |  | |  | 1 | 2 | | 3 | 4 | 5 | Post-treatment  30 |
| **Enrolment:**  Eligibility screen  Informed consent  Allocation |  | |  |  |  | |  |  |  |  |
|  | 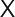 | |  |  |  | |  |  |  |  |
|  | 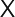 | |  |  |  | |  |  |  |  |
|  |  | | 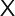 |  |  | |  |  |  |  |
| Intervention 1: CPAP + Usual physicaltherapy  Intervention 2: BIPAP + Usual Physicaltherapy  In  **Interventions:** |  | |  |  |  | |  |  |  |  |
|  |  | |  | 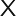 | 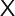 | | 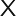 | 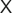 | 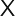 |  |
|  | Control: Usual physicaltherapy ptr | |  | 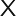X | 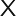 | | 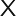 | 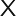 | 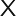 |  |
| **Assessments:**  *[lung function]*  *[pulmonary complications]*  *[time of during hospitalization]*  *[Quality of life]*  *[Submaximal capacity funcion]*  *[MIF]*  *[Gasometria]*  *[Outras]* | 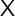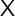  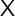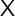  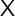  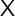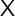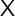 | |  |  |  | |  |  | 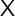  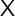  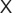  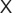  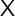    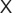 |  |
|  |  |  |  |  |  |  |  |  |  | 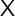 |
|  |  |  |  |  |  |  |  |  |  | 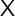 |
|  |  |  |  |  |  |  |  |  |  |  |
|  |  |  |  |  |  |  |  |  |  | 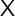 |
|  |  |  |  |  |  |  |  |  |  | 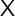 |
|  |  |  |  |  |  |  |  |  |  | 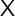 |
|  |  |  |  |  |  |  |  |  |  |  |
|  | 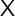 | |  |  |  | |  |  |  |  |

*[A escala de percepção global de mudança]*

**Tamanho da amostra (colocar média e DP para o cálculo)**: Amostra calculada utilizando nível de confiança de 95%, power de 80% e verificando-se desfecho pressões respiratórias máximas de trabalho referência na temática ^5^ utilizando-se da calculadora do site *openepi.com* chegamos ao valor de 62 pacientes.

**Recrutamento**: Homens e mulheres acima de 18 anos submetidos à cirurgia cardíaca na região metropolitana de Campina Grande no centro de referência em cardiologia. Demanda espontânea no serviço e busca ativa dentro do cadastro do setor. Amostragem com seleção não-probabilística por conveniência.

**Administração das intervenções**

**Alocação:**

*Geração da sequência*: Uma lista de números aleatórios gerada por computador será usada e uma sequência de randomização simples será criada pelo site *random.org*. Pacientes e terapeutas envolvidos não terão acesso à lista. Os pacientes serão randomizados com a razão de (1:1) por um investigador cego para identidade do paciente para um grupo intervenção CPAP + tratamento usual ou tratamento usual em fisioterapia. Após a randomização, a aplicação da VNI por avaliador cego e a equipe do estudo que vai coletar dados sobre os resultados do estudo desconhecerá atribuições do grupo de estudo. Todas as análises de dados também serão realizadas por pesquisador cego.

*Mecanismo de ocultação de alocação*: Envelopes opacos, selados e numerados em sequência serão utilizados pelo pesquisador para organizar a intervenção/controle com a equipe de profissionais e estudantes. Desse modo, haverá a disposição dos pacientes de forma randômica para os grupos 1, 2 e controle.

*Implementação*: Um pesquisador externo à pesquisa ficará responsável pela geração da alocação e organizará os envelopes selados e opacos.

**Cegamento**: Os participantes da pesquisa (pacientes) estarão cegos em relação à conduta, uma vez que utilizaremos o tratamento usual. Os profissionais que vão aplicar o tratamento não estarão cegos, uma vez que pela natureza da conduta associada à experiência dos terapeutas não conseguiríamos deixar de observar a utilização da VNI nos modos CPAP e BIPAP e as medidas da PEEP quando CPAP e IPAP e EPAP quando BIPAP serão necessários ajustes, impossibilitando o cegamento dos terapeutas. Os avaliadores dos desfechos estarão cegos, pois serão terapeutas diferentes dos avaliadores bem como os pesquisadores responsáveis pela análise dos dados, usando código de cores.

**Métodos de coleta de dados:** Inicialmente, o treinamento dos avaliadores será realizado para adequação da coleta, avaliação de resultados bem como gestão de dados iniciais, já para verificação da aplicação correta do protocolo preventivamente. Todos os instrumentos acima citados para coleta dos dados serão validados e calibrados no início do estudo ou durante o estudo quando for julgado necessário. Formulários para coleta de dados serão criados e disponibilizados em material suplementar. Durante toda a intervenção, os participantes serão conscientizados pelos terapeutas sobre a importância do tratamento e da pesquisa, bem como de manter a assiduidade e boa adaptação à conduta. Os participantes que por algum motivo tiverem um comportamento diferente do estabelecido nesse protocolo serão colocados em lista para que depois ocorra o seguimento com alguma técnica estatística (intenção de tratar). O planejamento para inserção e colocação dos dados será realizado semanalmente, com o pesquisador recolhendo as fichas de coleta de dados, bem como organizando os dados colocados em nuvem de armazenamento pelo monitoramento remoto dos pacientes, com o devido sigilo e cegamento.

**Métodos estatísticos: Os dados serão analisados usando o software estatístico IBM SPSS Statistics Base 25.0 para Windows. A normalidade das variáveis será avaliada pelo teste de Shapiro-Wilk. O teste t de Student para amostras pareadas será usado para comparar os dados antes e depois da intervenção. O efeito da VNI (CPAP ou BIPAP) mais tratamento usual de fisioterapia ao longo do tempo será comparado entre os grupos via análise de variância de duas vias (ANOVA). Além disso, usaremos a análise de covariância (ANCOVA), como análise de suporte, para comparar as diferenças entre os grupos após a intervenção, ajustando para valores dos respectivos resultados na linha de base. O tamanho do efeito será calculado usando o d de Cohen. As variáveis contínuas serão relatadas como um média ± desvio padrão (DP) e intervalo de confiança de 95% (IC 95%), e o valor categórico as variáveis serão apresentadas em frequências absolutas e percentuais. O nível de significância será fixado em 5% para todas as análises (p ≤ 0,05).**

**Monitoramento de dados: Em virtude da alta relevância da temática e da complexidade dos dados, um comitê de monitoramento de dados será formado. Composto pela professora orientadora do estudo e outro aluno auxiliar do departamento de Fisioterapia da UFRN a ser definido. Farão o trabalho de forma independente, com construção de relatórios preliminares de dados e sem conflito de interesses. Cabe ao comitê a análise pormenorizada dos dados iniciais com o poder inclusive de interromper o estudo antes do tempo previsto.**

**Danos: Um formulário específico para efeitos adversos será construído, disponibilizado em material suplementar e distribuído junto aos profissionais e estudantes que vão aplicar o protocolo. Serão armazenados e enviados semanalmente para o comitê de monitoramento de dados.**

**Auditoria: O comitê de monitoramento de dados terá programado alguma(s) visita(s) técnica ao hospital durante a aplicação do protocolo para verificação dos procedimentos.**

**Ética e divulgação: O presente protocolo será enviado ao comitê de ética em pesquisa da Universidade Estadual da Paraíba (UEPB).**

**Modificações no protocolo: Não serão realizadas modificações no protocolo após sua publicação na base de dados REBEC.**

**Consentimento ou assentimento: Todo participante deverá assinar um termo de consentimento livre e esclarecido (apêndice 1) logo no dia da internação no hospital. Um estudante ou profissional que for realizar a avaliação inicial solicitará ao paciente a sua assinatura.**

**Confidencialidade: Todos as informações pessoais não serão sob nenhuma hipótese divulgadas e os dados coletados serão mantidos em sigilo e apenas publicados de forma impessoal, garantindo a confidencialidade dos dados em revistas específicas científicas.**

**Declaração de interesses: Todos os pesquisadores envolvidos declaram não haver nenhum conflito de interesse.**

**Acesso aos dados: Os dados finais do trabalho ficarão de posse do pesquisador principal.**

**Tratamentos complementares e pós-ensaio: Caso exista algum dano ou efeito adverso decorrente da intervenção o paciente poderá reivindicar alguma conduta para compensação desse possível dano.**

**Política de divulgação: Após revisão por pares, deverá ser publicado em revista científica específica e conceituada.**

**REFERÊNCIAS BIBLIOGRÁFICAS**

1. A. Hossen, D. Jaju, M. Al-Abri, M. Mukaddirov, K. Al-Hashmi, Investigation of heart rate variability of patients undergoing coronary artery bypass grafting (CABG), Technol. Health Care 25 (2) (2017) 197–210, https://doi.org/10.3233/THC-161260.ATS. Committee on Proficiency Standards for Clinical Pulmonary Function Laboratories. (2002). ATS statement: guidelines for the six-minute walk test. Am J Respir Crit Care Med. 2002; 166:111-117.
2. A. Laizo, F.E. Delgado, G.M. Rocha, Complications that increase the time of hospital ization at ICU of patients submitted to cardiac surgery, Rev. Bras. Cir. Cardiovasc. 25 (2) (2010) 166–171, https://doi.org/10.1590/S0102-76382010000200007.Du H, Newton PJ, Salamonson Y, Carrieri-Kohlman VL, Davidson PM. A review of the six-minute walk test: its implication as a self-administered assessment tool. European journal of cardiovascular nursing. 2009; 8(1):2-8.
3. SHAKOURI, Seyed Kazem et al. Effect of respiratory rehabilitation before open cardiac surgery on respiratory function: a randomized clinical trial. Journal of cardiovascular and thoracic research, v. 7, n. 1, p. 13, 2015.Falk V, Baumgartner H, Bax JJ, De Bonis M, Hamm C, Holm PJ *et al*. ESC/EACTS Guidelines for the management of valvular heart disease. European Journal of Cardio-Thoracic Surgery. 2017; 52(4):616-664.
4. Valkenet K, de Heer F, Backx FC, et al. Effect of inspiratory muscle training before cardiac surgery in routine care. Phys Ther 2013; 93: 611–619.Kaufman R, Kuschnir MCC, Xavier RMA, Santos MA, Chaves RBM, Müller RE *et al*. Perfil epidemiológico na cirurgia de revascularização miocárdica. Rev Bras Cardiol. 2011: 24(6);369-76.
5. DOS SANTOS, Tamires Daros et al. Moderate-to-high intensity inspiratory muscle training improves the effects of combined training on exercise capacity in patients after coronary artery bypass graft surgery: A randomized clinical trial. International journal of cardiology, v. 279, p. 40-46, 2019.
6. American Thoracic Society/European Respiratory Society, ATS/ERS statement on respiratory muscle testing, Am. J. Respir. Crit. Care Med. 166 (4) (2002) 518– 624, https://doi.org/10.1164/rccm.166.4.518.
7. V.O. Carvalho, G.V. Guimarães, D. Carrara, F. Bacal, E.A. Bocchi, Validação da versão em português do Minnesota Living with Heart Failure Questionnaire, Arq. Bras. Cardiol. 93 (1) (2009) 39–44, https://doi.org/10.1590/S0066-782X2009000700008.
8. American Thoracic Society-ATS, Committee on proficiency standards for clinical pulmonary function laboratories. ATS statement: guidelines for the six-minute walk test, Am. J. Respir. Crit. Care Med. 166 (1) (2002) 111–117, https://doi.org/ 10.1164/ajrccm.166.1.at1102.

**APÊNDICES:**

# Apêndice A

TERMO DE CONSENTIMENTO LIVRE E ESCLARECIDO-TCLE

Pelo presente Termo de Consentimento Livre e Esclarecido eu, ________________________________________________, em pleno exercício dos meus direitos me disponho a participar da Pesquisa “**EFEITOS DA VENTILAÇÃO NÃO-INVASIVA COM DIFERENTES MODALIDADES EM PACIENTES SUBMETIDOS A CIRURGIA CARDÍACA: UM ENSAIO CLÍNICO RANDOMIZADO CONTROLADO**”. Declaro ser esclarecido e estar de acordo com os seguintes pontos: O trabalho **EFEITOS DA VENTILAÇÃO NÃO-INVASIVA COM DIFERENTES MODALIDADES EM PACIENTES SUBMETIDOS A CIRURGIA CARDÍACA: UM ENSAIO CLÍNICO RANDOMIZADO CONTROLADO** terá como objetivo: Realizar ensaio clínico randomizado verificar a eficácia da VNI em suas modalidades CPAP e BIPAP comparada à fisioterapia convencional no âmbito do índice de complicações pulmonares, função pulmonar e desfechos clínicos em pacientes submetidos a cirurgia cardíaca em hospital escolhido na cidade de Campina Grande, Paraíba. O voluntário da pesquisa será submetido a tratamento intervenção/controle envolvendo o uso da VNI com aparelho CPAP e ou BIPAP devidamente validado e calibrado, e uso dos cuidados usuais de fisioterapia do hospital Joao XXIII. Ao pesquisador caberá o desenvolvimento da pesquisa de forma confidencial; entretanto, quando necessário for, poderá revelar os resultados ao médico, indivíduo e/ou familiares, cumprindo as exigências da Resolução Nº. 466/12 do Conselho Nacional de Saúde/Ministério da Saúde. - O voluntário poderá se recusar a participar, ou retirar seu consentimento a qualquer momento da realização do trabalho ora proposto, não havendo qualquer penalização ou prejuízo para o mesmo. - Será garantido o sigilo dos resultados obtidos neste trabalho, assegurando assim a privacidade dos participantes em manter tais resultados em caráter confidencial. - Não haverá qualquer despesa ou ônus financeiro aos participantes voluntários deste projeto científico e não haverá qualquer procedimento que possa incorrer em danos físicos ou financeiros ao voluntário e, portanto, não haveria necessidade de indenização por parte da equipe científica e/ou da Instituição responsável. - Qualquer dúvida ou solicitação de esclarecimentos, o participante poderá contatar a equipe científica no número (083) 988998813 com Eder Rodrigues Araújo. - Ao final da pesquisa, se for do meu interesse, terei livre acesso ao conteúdo da mesma, podendo discutir os dados, com o pesquisador, vale salientar que este documento será impresso em duas vias e uma delas ficará em minha posse. - Desta forma, uma vez tendo lido e entendido tais esclarecimentos e, por estar de pleno acordo com o teor do mesmo, dato e assino este termo de consentimento livre e esclarecido.

_______________________________

Assinatura do pesquisador responsável

_______________________________

Assinatura do Participante
