# Supplementary material for: Effects of Non-Invasive Ventilation with different modalities in patients undergoing heart surgery: Protocol for a randomized controlled clinical trial
Source: PLoS One. 2024 Jun 18;19(6):e0304569. doi: 10.1371/journal.pone.0304569 (PMC11185470; doi:10.1371/journal.pone.0304569)
Supplement: S2 File — (DOC) [file pone.0304569.s004.doc]

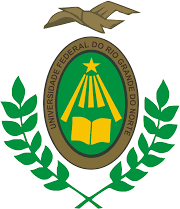


**FEDERAL UNIVERSITY OF RIO GRANDE DO NORTE**

**HEALTH SCIENCES CENTER**

**POSTGRADUATE PROGRAM IN PHYSIOTHERAPY**

**SUBJECT: CLINICAL TRIAL PROJECTS IN PHYSIOTHERAPY AND PROTOCOL DEVELOPMENT**

**TEACHER: DR PATRÍCIA ANGÉLICA DE MIRANDA SILVA NOGUEIRA**

**STUDENT: EDER RODRIGUES ARAÚJO**

**EFFECTS OF NON-INVASIVE VENTILATION WITH DIFFERENT MODALITIES IN PATIENTS UNDERGOING HEART SURGERY: A RANDOMIZED CONTROLLED CLINICAL TRIAL**

NATAL - RN

2022

# GENERAL INFORMATION

# This protocol is part of Eder Rodrigues Araújo's doctoral thesis, under the guidance of Prof. Patrícia Angélica de Miranda Silva Nogueira and will be registered in the Brazilian Clinical Trials Registry (REBEC). This is version No. 1 – 11/22/2022.

# Funding: This study will be financed in part by the Coordination for the Improvement of Higher Education Personnel - Brazil (CAPES) - Financial Code 001. The study will also have a partnership with the health technology center (NUTES-UEPB) to finance wearable devices remote monitoring related to the research.

# Roles and responsibilities:

# Eder Rodrigues Araújo, UFRN, doctoral student, eder.rodrigues.araujo@gmail.com main author of the protocol, will participate in the training of physiotherapists to apply the protocol and subsequent analyses.

# Patrícia Angélica de Miranda Silva Nogueira, UFRN, PhD professor, idpa02@hotmail.com, advisor, co-author of the protocol, will participate in the technical supervision of the writing of the protocol, the analysis and discussions of the study and the coordination of the data monitoring committee.

# NUTES/UEPB: Technology institution at the State University of Paraíba that has a partnership with the UFRN health measurement and evaluation laboratory. They will participate in the acquisition and availability of smartwatches for remote monitoring of patients' vital signs.

# ABSTRACT

# The thoracic surgical procedure causes a reduction in respiratory muscle strength. To restore it, some strategies must be used. Physiotherapy uses resources and techniques, such as stimulation of deep breathing, stimulation of coughing, use of incentive spirometers, mobilization and ambulation. However, sometimes these resources and techniques are not sufficient, and additional resources, such as the use of NIV, are employed19. Non-invasive positive pressure ventilation (NPPV) has been used to accelerate the recovery of lung function as well as prevent and treat postoperative pulmonary complications 16. NIV reduces the risk of ventilator-associated complications due to its non-invasive characteristics. Thus, NIV has been adopted to prevent complications after extubation in postoperative patients18. The objective of this work is to carry out a randomized clinical trial and verify the effectiveness of NIV compared to conventional physiotherapy in terms of the rate of pulmonary complications in patients undergoing cardiac surgery in a chosen hospital in the city of Campina Grande, Paraíba. randomized controlled, double blind (patient, analyst) that proposes the intervention with use in the BIPAP group of the NIV BIPAP modality in a group with usual physiotherapy care and in the CPAP group of the NIV CPAP modality in a group with usual physiotherapy care and the group control with just usual physical therapy care. The outcomes observed will be: assessment between NIV modalities, spirometry, lung capacity and vital signs. Secondary outcomes included are degree of satisfaction using the global perception of change scale, and MIF – measure of functional independence and blood gas parameters. It is hoped that the results of this research can contribute to the verification of NIV in surgical heart disease patients in clinical practice.

**Introduction**

In the United States, more than 2,200 Americans die every day from cardiovascular causes, and people over the age of 65 are responsible for almost 80% of these deaths. The American Heart Association reports that older adults are also responsible for almost 75% of cardiovascular diseases. The national burden of morbidity and mortality caused by cardiovascular events remains high, and there is a search for efficient and effective preventive measures.5

In patients with cardiovascular disease, including valvular disease, current guidelines advise considering exercise capacity for diagnosis and treatment planning.6,7 Additionally, coronary artery disease is the leading cause of death worldwide and patients undergoing surgery of myocardial revascularization (CABG) constitute the highest risk group.8

The constant increase in patients with heart disease denotes the need to generate new devices adaptable to the reality of these patients. Progressive measures are needed to promote better services for this risk group. 9.10

Respiratory complications continue to be one of the main causes of morbidity in cardiac surgery patients17. The etiology of pulmonary complications results from a multifactorial process. Surgical factors such as use of extracorporeal circulation (CPB), anesthesia, surgery time, mechanical ventilation time, pleural opening, alteration of the phrenic nerve, use of the mammary artery in myocardial revascularization surgery, pain in the sternal surgical wound and in surgical drains lead to a decrease in functional residual capacity and an increase in intrapulmonary shunt. Furthermore, preoperative factors related to the patient, such as pre-existing lung diseases, smoking, advanced age, poor nutritional health, among others, predispose to complications. 19

Certain measures are used during the postoperative period of cardiac surgeries in an attempt to minimize pulmonary complications, such as adequate analgesia, oxygen therapy, and physiotherapy. Physiotherapy utilizes resources and techniques like deep breathing stimulation, cough stimulation, the use of incentive spirometers, mobilization, and ambulation. However, sometimes these resources and techniques are not sufficient, and additional measures, such as the use of NIV, are employed.

Non-invasive positive pressure ventilation (NPPV) has been used to expedite the recovery of pulmonary function, as well as to prevent and treat postoperative pulmonary complications.

NIV is a support for spontaneous ventilation. Its use as a prophylactic measure aims to reduce the incidence of endotracheal intubation, length of hospital stay, and prevent pulmonary complications. However, even with randomized clinical trials (RCTs) and systematic reviews, there is no consensus in the literature regarding its use as a prophylactic measure after cardiac surgery.

Postoperative pulmonary complications are the most common due to perioperative factors as well as the close anatomical and functional relationship between the heart and the lungs. A prevalence of these complications ranging from 5% to 20% has been documented, and they are associated with increased postoperative morbidity and mortality.

Non-invasive ventilation (NIV) denotes the administration of ventilatory support without the use of an invasive artificial airway (endotracheal tube or tracheostomy tube). NIV supports breathing in patients with various conditions, such as cardiogenic pulmonary edema, exacerbations of chronic obstructive pulmonary disease, and those who have suffered closed chest trauma. NIV improves gas exchange, assists with breathing, and reduces the need for positive pressure support intubation. NIV reduces the risk of complications associated with mechanical ventilation due to its non-invasive characteristics. Therefore, NIV has been adopted to prevent complications after extubation in postoperative patients.

Continuous positive airway pressure (CPAP) and bilevel positive airway pressure (BiPAP) are the most common methods of NIV.

Therefore, with the aim of integrating these themes and providing more support for the decision on the best NIV modality in cardiac surgery patients, the objective of this research project is to conduct a randomized clinical trial and assess the effectiveness of NIV in its CPAP and BiPAP modalities compared to conventional physiotherapy in terms of pulmonary complications, pulmonary function, and clinical outcomes in patients undergoing cardiac surgery at a selected hospital in the city of Campina Grande, Paraíba.

# OBJECTIVE

To carry out a randomized clinical trial to verify the effectiveness of NIV in its CPAP and BIPAP modalities compared to conventional physiotherapy in terms of the rate of pulmonary complications, pulmonary function and clinical outcomes in patients undergoing cardiac surgery in a chosen hospital in the city of Campina Grande, Paraíba.

**Specific objectives**

Develop a platform for integrating all clinical data from patients undergoing cardiac surgery;

• Describe the profile of heart disease patients undergoing cardiac surgery;

• Facilitate the work carried out by professionals involved in the care of heart disease patients undergoing surgery;

• Profile patients in relation to spirometry, 6-minute walk test, satisfaction, vital signs, MIF and blood gas parameters.

# Study design

# The study protocol was conducted following the Standard Protocol Items: Recommendations for Interventional Trials (SPIRIT) checklist. This is a randomized controlled, double-blind, single-center clinical trial, in which participants and outcome assessors were blinded. The study will be submitted to the ethics committee of the State University of Paraíba (UEPB) and carried out in accordance with the Declaration of Helsinki. All participants must provide written informed consent prior to participation, and the study will be registered on the REBEC platform. All participants must provide written informed consent prior to participation. Participants will be divided into three groups (cpap group – CPAP + usual physiotherapy care, bipap group – BIPAP + usual physiotherapy care and control – usual physiotherapy care), in a 1:1 allocation and superiority ratio.METODOS

**Participants, interventions and outcomes**

**Study location**

The project will be carried out at Hospital João XXIII in the city of Campina Grande, Paraíba, Brazil. It is a private hospital affiliated with the SUS network, a reference in cardiology and especially in cardiac surgery for much of the region of the state and surrounding states. He presents an average of 8 heart surgeries on his weekly program. Assessment and intervention will take place in the ICU and ward. The Institutional Authorization Term for the legal conduct of the research has already been signed (APPENDIX A).

**Eligibility Criteria**

**Inclusion criteria:** Patients undergoing cardiac surgery, aged over 18 years, stable from a hemodynamic point of view (controlled blood pressure, normocardic – conscious and oriented and cooperative) without medical restrictions to undergo treatment**.**

**Exclusion criteria**: Presence of uncontrolled cardiac arrhythmias, previous neuromuscular disease, labyrinthitis and some orthopedic/skeletal muscle restrictions. Unstable angina, stage 3 hypertension (SBP ≥ 180 mmHg and/or DBP ≥ 110 mmHg at rest), HR > 120 bpm at rest, systemic arterial hypotension with clinical repercussions (SBP ≤ 90 mmHg and/or DBP ≤ 60 mmHg), arrhythmias uncontrolled (Examples: total atrioventricular block, type 2 2nd degree atrioventricular block, atrial fibrillation, sustained ventricular tachycardia), aortic dissection.

**Post-randomization exclusion criteria:** In the case of undergoing any treatment in the hospital that contraindicates participation in the study (pacemaker implantation, pneumothorax requiring chest drainage, for example), death or other decompensation unrelated to the research and return to the ICU..

**Criteria for non-adherence to the intervention**: Patient who does not perform 3 or more sessions in a row, who withdrew from participating in the study and who presents a cardiac arrhythmia (atrial fibrillation, bradycardia) for two sessions even after medication used to contain it and a rest break.

**Non-retention criteria: Patient who does not undergo one of the reevaluations.**

**Professional eligibility criteria: Physiotherapists from the hospital team and physiotherapy students with prior training in the research protocol.**

**INTERVENTION**

**The intervention will initially take place in the specific ward that receives patients in the postoperative period of cardiac surgery. Participants will be recruited from the patient's admission on the day before surgery, during which they will undergo pre-operative assessment using the study criteria. On this first day, the evaluation will consist of anamnesis and physical examination, focusing on the history of cardiac disorders, followed by measurements inherent to the protocol. Manovacuometry, chest x-ray, quality of life questionnaire, walking test and spirometry. The assessment can be carried out in two stages, if there are other demands on the patient during their hospital stay.**

**Physiotherapists and physiotherapy students will be trained in relation to the research protocol, comprising two face-to-face meetings with demonstrations and teaching material. For both the intervention and control groups, the therapists will be the same.**

**The intervention is scheduled to begin in March 2023, after approval by the ethics and research committee and adjustments to bureaucratic dealings with the hospital.**

**Control group (Usual care):** **Patients undergoing usual physiotherapy treatment, consisting of a kinesiotherapy protocol. Patients will undergo treatment twice a day, interspersing treatment already started in the ICU environment and progressing to the ward. In detail, we have a division by days and environments, observing the general characteristic of the patient being discharged from the ICU on the 2nd postoperative day and that day 1 represents the first postoperative day:**

**DAY 1 (ICU): Diaphragmatic breathing exercises – 1 x 10; Cough stimulation; Diaphragmatic breathing exercises associated with upper limbs – Shoulder flexion/extension 2 x 10 up to 90 degrees; Shoulder abduction 1 x 10 (remove to avoid pain); Diaphragmatic breathing exercises associated with lower limbs – Thigh flexion 1 x 10; Dorsiflexion/plantar flexion 1 x 10.**

**DAY 2 (ICU): Diaphragmatic breathing – 1 x 10; Cough stimulation; Breathing exercises associated with upper limbs – Shoulder flexion 2 x 10; Breathing exercises associated with lower limbs – Thigh flexion 1 x 10; Dorsiflexion/plantar flexion 1 x 10; Cycle ergometer 3 min; Breathing exercise with SMI 1x10**

**DAY 3 (WARD): Diaphragmatic breathing – 1 x 10; Cough stimulation; Breathing exercises associated with upper limbs – Shoulder flexion 2 x 10; Breathing exercises associated with lower limbs – Thigh flexion 1 x 10; Dorsiflexion/plantar flexion 1 x 10; Walking 5 min.**

**DAY 4 (WARD): Diaphragmatic breathing – 1 x 10; Cough stimulation; Walking 10 min.**

**DAY 5 (WARD): Diaphragmatic breathing – 1 x 10; Cough stimulation; Walking 15 min.**

**Intervention group 1 (CPAP): They will undergo the same care as the control group, adding NIV with nasal CPAP 10cmH2O for 1 hour using a _________________brand ___________ device, during the 5 days of hospitalization, both in the ICU and in the ward. The frequency of sessions will be two (2) per day, in the morning and afternoon. Flexibility in the schedule for carrying out the procedure is also planned, since in a hospital environment the patient can often undergo exams and other procedures that can make it difficult to apply the protocol at the initially scheduled time. After the fifth day, the patient will be reevaluated with the same instruments reported.**

**Intervention group 2 (BIPAP): They will undergo the same care as the control group, adding NIV with nasal BIPAP with IPAP of 13cmH2O and EPAP 8 cmH2O for 1 hour, using a _________________brand ___________ device, during the 5 days of hospitalization, both in ICU and in the ward. The frequency of sessions will be two (2) per day, in the morning and afternoon. Flexibility in the schedule for carrying out the procedure is also planned, since in a hospital environment the patient can often undergo exams and other procedures that can make it difficult to apply the protocol at the initially scheduled time. After the fifth day, the patient will be reevaluated with the same instruments reported.**

**Adherence: During the evaluation and conduct in daily sessions, the therapist will be instructed to carry out awareness, guidance, and description of the purpose of the research, in order to obtain maximum adherence to the protocol. The adherence assessment will be carried out with direct contact between the researcher and the physiotherapists and study students in a messaging application group previously formed specifically for this purpose. Adhesion monitoring will also include frequent visits by the researcher at the time of application of the protoocolar treatment.**

**Concomitant care: Patients who require a longer stay in the ICU with the use of larger devices for prolonged stay on non-invasive ventilation or even a return to IMV during the study period will be considered non-adherent from that period onwards, and thus the use of intention to treat is anticipated.**

**Follow-up: Scheduled for one (1) month after hospital discharge, the patient will be re-evaluated after the medical consultation after cardiac surgery.**

**Primary:**

***Lung capacity: Spirometry will check vital capacity (VC), forced expiratory vital capacity (FVC), forced expiratory volume in the first second (FEV1) and the FEV1/FVC ratio. The metric considered will also be seen before and after treatment, also checking the delta. We will use Contec SP80B portable spirometer equipment.***

***Length of hospital stay: Outcome variable verified by the absolute number of days of hospitalization. Important outcome for checking hospital costs and postoperative complications, mainly.***

***Pulmonary complications: Patients will have their preoperative radiographs compared with the chest x-ray on the day of hospital discharge, as well as reports of pulmonary complications during hospitalization will be verified, such as the presence of atelectasis, pleural effusion without the need for chest drainage, pleural effusion with history of chest tube drainage. The evaluation will be carried out by the researcher, checking reports issued by the hospital's radiology team. The metric considered will be the presence of complications, checking the before and after.***

**Secondary:**

**Quality of life: The Portuguese version of the Minnesota Living with Heart Failure Questionnaire (MLHFQ) will be used to assess the quality of life of patient 7. The questionnaire has a final metric in score and the delta in variation during this treatment period will be evaluated.**

**Submaximal functional capacity: The 6MWT will be performed in accordance with ATS 8 guidelines. The greatest six-minute walking distance (6MWD) was considered for analysis and compared with the prediction. The metric considered will also be seen before and after treatment, also checking the delta. The 6-minute walk test will be carried out based on the initial protocol developed according to international guidelines on the subject, in an appropriate location provided by the hospital, with all the necessary structure and demarcation. After registering the patient and placing the smartwatch, the researcher will submit the patient to the test. The data is now collected automatically and placed on the aforementioned platform, with all vital signs depending on the test time. Performed preoperatively and on the sixth day.**

**The global perception of change scale will also be applied on the sixth day, according to the adaptation by Domingues, 2011.**

**The functional independence measure (FIM) will be carried out preoperatively and on the sixth day, using the questionnaire adapted from Borges, 2006.**

**The blood gas data will be analyzed and verified in the patient's medical record in the immediate postoperative period, before the patient starts the protocol.**

**Other variables will be collected by analyzing the patient's medical records: Cardiopulmonary bypass time, length of hospital stay, type of surgery, personal history, preoperative ejection fraction, respiratory complications during hospitalization, weight, height and BMI, sex and age .**

**SAMPLE CHARACTERIZATION MEASURES – MONITORING: Measurement and monitoring of vital signs throughout the treatment.**

**Risks and adverse effects: During treatment, there are minimal risks of cardiac arrhythmias, drops in saturation levels, dyspnea, bronchospasm, dizziness, syncope, hypotension or arterial hypertension, changes in heart rate or chest pain. During the entire period, the patient will be monitored for vital signs using the Garmin Forerruner 945 Smartwatch with remote connection and measurement of maximum oxygen consumption (VO2), oxygen saturation (SatO2) and heart rate (HR) with its own validation for these variables. If these situations mentioned above occur, the patient will have treatment interrupted to compensate for the condition and reevaluate to safely return to research.**

**Adverse events: Any events related to cardiovascular disorders will be reported using the smartwatch. Throughout the protocol, the patient will be contacted and asked about any changes outside the treatment period, by patient self-report.**

# Fluxogram

|  |  | Pre-treatment  Post-allocation | | | | | | | | |
| --- | --- | --- | --- | --- | --- | --- | --- | --- | --- | --- |
|  |  | | |  | | 4 | | | |  |
| **TIMEPOINT*(days)** |  | |  | 1 | 2 | | 3 | 4 | 5 | Post-treatment  30 |
| **Enrolment:**  Eligibility screen  Informed consent  Allocation |  | |  |  |  | |  |  |  |  |
|  | 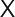 | |  |  |  | |  |  |  |  |
|  | 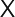 | |  |  |  | |  |  |  |  |
|  |  | | 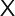 |  |  | |  |  |  |  |
| Intervention 1: CPAP + Usual physicaltherapy  Intervention 2: BIPAP + Usual Physicaltherapy  In  **Interventions:** |  | |  |  |  | |  |  |  |  |
|  |  | |  | 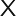 | 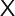 | | 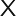 | 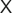 | 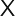 |  |
|  | Control: Usual physicaltherapy ptr | |  | 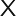X | 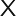 | | 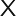 | 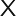 | 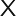 |  |
| **Assessments:**  *[lung function]*  *[pulmonary complications]*  *[time of during hospitalization]*  *[Quality of life]*  *[Submaximal capacity funcion]*  *[MIF]*  *[Gasometria]*  *[Outras]* | 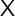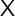  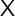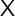  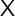  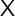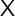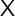 | |  |  |  | |  |  | 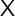  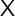  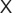  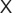  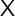    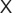 |  |
|  |  |  |  |  |  |  |  |  |  | 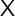 |
|  |  |  |  |  |  |  |  |  |  | 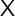 |
|  |  |  |  |  |  |  |  |  |  |  |
|  |  |  |  |  |  |  |  |  |  | 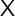 |
|  |  |  |  |  |  |  |  |  |  | 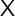 |
|  |  |  |  |  |  |  |  |  |  | 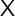 |
|  |  |  |  |  |  |  |  |  |  |  |
|  | 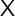 | |  |  |  | |  |  |  |  |

*[A escala de percepção global de mudança]*

Sample size (enter mean and SD for calculation): Sample calculated using a confidence level of 95%, power of 80% and checking the outcome of maximum working respiratory pressures reference in theme 5 using the calculator on the openepi website. with we reached the value of 62 patients.

Recruitment: Men and women over 18 years of age undergoing cardiac surgery in the metropolitan region of Campina Grande at the cardiology reference center. Spontaneous demand for the service and active search within the sector registry. Sampling with non-probabilistic selection for convenience.

Administration of interventions

Allocation**:** *Sequence generation: A computer-generated list of random numbers will be used and a simple randomization sequence will be created via random.org. Patients and involved therapists will not have access to the list. Patients will be randomized with a ratio of (1:1) by an investigator blinded to patient identity to a CPAP intervention + usual care or usual care physiotherapy group. After randomization, the application of NIV by a blinded evaluator and the study team who will collect data on the study results will be blinded to study group assignments. All data analyzes will also be carried out by a blind researcher.*

*Allocation concealment mechanism: Opaque, sealed and sequentially numbered envelopes will be used by the researcher to organize the intervention/control with the team of professionals and students. In this way, patients will be randomly assigned to groups 1, 2 and control.*

*Implementation: A researcher external to the research will be responsible for generating the allocation and will organize the sealed and opaque envelopes.*

Blinding: The research participants (patients) will be blinded to the treatment since we will be using the usual treatment. The professionals who will administer the treatment will not be blinded because, due to the nature of the procedure associated with the therapists' experience, it would be impossible to conceal the use of NIV in CPAP and BIPAP modes, as well as the PEEP measurements when CPAP and IPAP and EPAP when BIPAP adjustments are required, making blinding of the therapists unfeasible. Outcome assessors will be blinded, as they will be different therapists from the assessors, and the researchers responsible for data analysis will use a color-coding system for blinding.

Data Collection Methods: Initially, training of the assessors will be conducted to ensure proper data collection, outcome assessment, and initial data management to verify the correct application of the protocol proactively. All the instruments mentioned above for data collection will be validated and calibrated at the beginning of the study or during the study when deemed necessary. Data collection forms will be created and made available in supplementary materials. Throughout the intervention, participants will be educated by the therapists about the importance of treatment and research, as well as maintaining attendance and good adherence to the protocol. Participants who, for any reason, exhibit behavior different from what is established in this protocol will be included in a follow-up with a statistical technique (intention-to-treat). Data entry and management will be performed weekly, with the researcher collecting data collection forms and organizing the data stored in cloud storage through remote patient monitoring, ensuring confidentiality and blinding.

Statistical Methods: The data will be analyzed using IBM SPSS Statistics Base 25.0 software for Windows. Normality of variables will be assessed using the Shapiro-Wilk test. The paired t-test will be used to compare data before and after the intervention. The effect of NIV (CPAP or BIPAP) plus usual physiotherapy treatment over time will be compared between groups using a two-way analysis of variance (ANOVA). Additionally, we will use analysis of covariance (ANCOVA) as a supporting analysis to compare differences between groups after the intervention, adjusting for baseline values of respective outcomes. Effect size will be calculated using Cohen's d. Continuous variables will be reported as mean ± standard deviation (SD) and 95% confidence interval (CI), and categorical variables will be presented as absolute frequencies and percentages. The significance level will be set at 5% for all analyses (p ≤ 0.05).

Data Monitoring: Given the high relevance of the topic and the complexity of the data, a data monitoring committee will be formed. It will consist of the study's supervising professor and another student assistant from the Department of Physiotherapy at UFRN, to be determined. They will work independently, generating preliminary data reports without conflicts of interest. The committee is responsible for a detailed analysis of initial data and has the authority to prematurely stop the study.

Harms: A specific form for adverse effects will be developed, made available in supplementary materials, and distributed to professionals and students who will apply the protocol. Adverse events will be stored and sent weekly to the data monitoring committee.

Audit: The data monitoring committee will schedule one or more technical visits to the hospital during the protocol application to verify procedures.

Ethics and Disclosure: This protocol will be submitted to the research ethics committee of the State University of Paraíba (UEPB).

Protocol Modifications: No modifications will be made to the protocol after its publication in the REBEC database.

Consent or Assent: Every participant must sign an informed consent form (Appendix 1) on the day of hospital admission. A student or professional conducting the initial assessment will request the patient's signature.

Confidentiality: All personal information will not be disclosed under any circumstances, and collected data will be kept confidential and only published in an impersonal manner, ensuring data confidentiality in specific scientific journals.

Declaration of Interests: All researchers involved declare no conflicts of interest.

Data Access: The final data from the study will be in the possession of the principal researcher.

Complementary and Post-Trial Treatments: If there is any harm or adverse effect resulting from the intervention, the patient may seek compensation for such harm.

Disclosure Policy: After peer review, the results will be published in a specific and reputable scientific journal.

**BIBLIOGRAPHIC REFERENCES**

1. A. Hossen, D. Jaju, M. Al-Abri, M. Mukaddirov, K. Al-Hashmi, Investigation of heart rate variability of patients undergoing coronary artery bypass grafting (CABG), Technol. Health Care 25 (2) (2017) 197–210, https://doi.org/10.3233/THC-161260.ATS. Committee on Proficiency Standards for Clinical Pulmonary Function Laboratories. (2002). ATS statement: guidelines for the six-minute walk test. Am J Respir Crit Care Med. 2002; 166:111-117.
2. A. Laizo, F.E. Delgado, G.M. Rocha, Complications that increase the time of hospital ization at ICU of patients submitted to cardiac surgery, Rev. Bras. Cir. Cardiovasc. 25 (2) (2010) 166–171, https://doi.org/10.1590/S0102-76382010000200007.Du H, Newton PJ, Salamonson Y, Carrieri-Kohlman VL, Davidson PM. A review of the six-minute walk test: its implication as a self-administered assessment tool. European journal of cardiovascular nursing. 2009; 8(1):2-8.
3. SHAKOURI, Seyed Kazem et al. Effect of respiratory rehabilitation before open cardiac surgery on respiratory function: a randomized clinical trial. Journal of cardiovascular and thoracic research, v. 7, n. 1, p. 13, 2015.Falk V, Baumgartner H, Bax JJ, De Bonis M, Hamm C, Holm PJ *et al*. ESC/EACTS Guidelines for the management of valvular heart disease. European Journal of Cardio-Thoracic Surgery. 2017; 52(4):616-664.
4. Valkenet K, de Heer F, Backx FC, et al. Effect of inspiratory muscle training before cardiac surgery in routine care. Phys Ther 2013; 93: 611–619.Kaufman R, Kuschnir MCC, Xavier RMA, Santos MA, Chaves RBM, Müller RE *et al*. Perfil epidemiológico na cirurgia de revascularização miocárdica. Rev Bras Cardiol. 2011: 24(6);369-76.
5. DOS SANTOS, Tamires Daros et al. Moderate-to-high intensity inspiratory muscle training improves the effects of combined training on exercise capacity in patients after coronary artery bypass graft surgery: A randomized clinical trial. International journal of cardiology, v. 279, p. 40-46, 2019.
6. American Thoracic Society/European Respiratory Society, ATS/ERS statement on respiratory muscle testing, Am. J. Respir. Crit. Care Med. 166 (4) (2002) 518– 624, https://doi.org/10.1164/rccm.166.4.518.
7. V.O. Carvalho, G.V. Guimarães, D. Carrara, F. Bacal, E.A. Bocchi, Validação da versão em português do Minnesota Living with Heart Failure Questionnaire, Arq. Bras. Cardiol. 93 (1) (2009) 39–44, https://doi.org/10.1590/S0066-782X2009000700008.
8. American Thoracic Society-ATS, Committee on proficiency standards for clinical pulmonary function laboratories. ATS statement: guidelines for the six-minute walk test, Am. J. Respir. Crit. Care Med. 166 (1) (2002) 111–117, https://doi.org/ 10.1164/ajrccm.166.1.at1102.

**APPENDICES:**

**Appendix A**

INFORMED CONSENT FORM (ICF)

Through this Informed Consent Form, I, ________________________________________________, fully exercising my rights, willingly agree to participate in the research study titled "EFFECTS OF NON-INVASIVE VENTILATION WITH DIFFERENT MODALITIES IN PATIENTS UNDERGOING CARDIAC SURGERY: A RANDOMIZED CONTROLLED CLINICAL TRIAL." I acknowledge that I am well-informed and agree with the following points:

The study "EFFECTS OF NON-INVASIVE VENTILATION WITH DIFFERENT MODALITIES IN PATIENTS UNDERGOING CARDIAC SURGERY: A RANDOMIZED CONTROLLED CLINICAL TRIAL" aims to conduct a randomized clinical trial to investigate the efficacy of Non-Invasive Ventilation (NIV) using CPAP and BiPAP modalities compared to conventional physiotherapy in terms of pulmonary complications, lung function, and clinical outcomes in patients undergoing cardiac surgery at a selected hospital in Campina Grande, Paraíba, Brazil.

As a research participant, I will undergo intervention/control treatment involving the use of properly validated and calibrated CPAP and/or BiPAP devices for NIV, in addition to receiving the usual physiotherapy care provided by Joao XXIII Hospital. The researcher is responsible for conducting the study confidentially. However, if necessary, the researcher may disclose the results to the physician, individual, and/or family members, in accordance with the requirements of Resolution No. 466/12 from the National Health Council/Ministry of Health.

I have the right to refuse to participate or withdraw my consent at any point during the execution of the proposed study, without facing any penalties or disadvantages.

The confidentiality of the obtained results will be ensured, maintaining the privacy of participants by keeping such results strictly confidential.

There will be no expenses or financial burden on voluntary participants in this scientific project, and no procedures that could lead to physical or financial harm to the participant will be conducted. Therefore, there is no requirement for compensation from the scientific team and/or the responsible institution.

In case of any doubts or requests for clarifications, I may contact the research team at the number (083) 988998813, with Eder Rodrigues Araújo.

Upon completion of the research, if I wish, I will have access to the content of the study and can discuss the data with the researcher. It is important to note that this document will be printed in two copies, with one copy staying in my possession.

By reading and understanding these explanations and fully agreeing with their contents, I sign and date this Informed Consent Form.

Signature of Responsible Researcher

Signature of Participant
